# Inverse Association between Glycated Albumin and Insulin Secretory Function May Explain Higher Levels of Glycated Albumin in Subjects with Longer Duration of Diabetes

**DOI:** 10.1371/journal.pone.0108772

**Published:** 2014-09-29

**Authors:** Yong-ho Lee, Mi Hyang Kown, Kwang Joon Kim, Eun Young Lee, Daham Kim, Byung-Wan Lee, Eun Seok Kang, Bong Soo Cha, Hyun Chul Lee

**Affiliations:** 1 Department of Internal Medicine, Yonsei University College of Medicine, Seoul, South Korea; 2 Severance Hospital, Seoul, South Korea; 3 Severance Executive Healthcare Clinic, Yonsei University Health System, Seoul, South Korea; 4 Department of Biochemistry and Molecular Biology, Brain Korea 21 PLUS Project for Medical Science, Yonsei University College of Medicine, Seoul, South Korea; Faculty of Biology, Spain

## Abstract

**Background:**

Glycated albumin (GA) has been increasingly used as a reliable index for short-term glycemic monitoring, and is inversely associated with β-cell function. Because the pathophysiologic nature of type 2 diabetes (T2D) is characterized by progressive decline in insulin secretion, the aim was to determine whether GA levels were affected by diabetes duration in subjects with T2D.

**Methods:**

To minimize the effect of glucose variability on GA, subjects with stably maintained HbA_1c_ levels of <0.5% fluctuation across 6 months of measurements were included. Patients with newly diagnosed T2D (*n* = 1059) and with duration>1 year (*n* = 781) were recruited and categorized as New-T2D and Old-T2D, respectively. Biochemical, glycemic, and C-peptide parameters were measured.

**Results:**

GA levels were significantly elevated in HbA_1c_-matched Old-T2D subjects compared to New-T2D subjects. Duration of diabetes was positively correlated with GA, whereas a negative relationship was found with C-peptide increment (ΔC-peptide). Among insulin secretory indices, dynamic parameters such as ΔC-peptide were inversely related to GA (*r* = −0.42, *p*<0.001). Multiple linear regression analyses showed that duration of diabetes was associated with GA (standardized β coefficient [STDβ] = 0.05, *p*<0.001), but not with HbA_1c_ (STDβ = 0.04, *p*<0.095). This association disappeared after additional adjustment with ΔC-peptide (STDβ = 0.02, *p* = 0.372), suggesting that β-cell function might be a linking factor of close relationship between duration of diabetes and GA values.

**Conclusions:**

The present study showed that GA levels were significantly increased in subjects with longer duration T2D and with decreased insulin secretory function. Additional caution should be taken when interpreting GA values to assess glycemic control status in these individuals.

## Introduction

The United Kingdom Prospective Diabetes Study (UKPDS) [1] and Kumamoto Study [2] demonstrated that intensive glycemic control was essential for individuals with type 2 diabetes (T2D) to reduce risks of morbidity and mortality from diabetes-related complications and cardiovascular diseases. In line with these results, the purpose of glucose monitoring with glycemic indices, in conjunction with lifestyle modification, and pharmacological anti-diabetic drugs in diabetic subjects, is to achieve and maintain optimal blood glucose levels [3]. Clinical data of HbA_1c_ levels have been shown to predict average glycemic status over several months, and to predict diabetic complications [1], [4]; therefore the American Diabetes Association recommended routine HbA_1c_ testing in all diabetic patients as an initial assessment, and then as a part of continuing care [3].

By overcoming the unmet need of well-established or gold standard glycemic index of HbA_1c_ [3], glycated albumin (GA) measurements have been increasingly used as another reliable index for 3-week intermediate glycation monitoring, and GA measurements are further preferred for use in several pathologic conditions, including anemia and chronic kidney disease (CKD) [5]–[7]. In addition, GA is closely associated with insulin secretory function of β-cells [8], [9]. The pathophysiologic nature of T2D is characterized by a progressive decline in pancreatic β-cell function, with deterioration and insulin resistance, resulting in the failure of insulin secretion from islet cells. As duration of diabetes increases, β-cell functions are well known to gradually decline, which may affect the levels of GA. Considering the greater association of GA with decreasing pancreatic β-cell functions than with HbA_1c_ levels in subjects with T2D, the relationship between GA levels and diabetic duration may differ from what has been previously accepted. However, to date there is no report on the relationship among GA, β-cell function and duration of diabetes in subjects with T2D. On these bases, the aim of our study was to investigate whether GA levels are elevated in subjects with long duration of diabetes in close connection with their insulin secretory capacity.

## Methods

### Subjects and data collection

In this cross-sectional study, patients with T2D who visited the Diabetes Center at Severance Hospital, Seoul, South Korea between January 2009 and December 2012 were enrolled in the Diabetes Registry of Severance Hospital, and most of the subjects have been described in our previous studies [9]–[11]. The study subjects were classified into two groups according to the duration of diabetes. New-onset type 2 diabetes was defined as patients whose duration of diabetes was ≤1 year (New-T2D), and old T2D included patients with duration of diabetes >1 year (Old-T2D). Based on electronic medical records, a total of 1840 subjects satisfied the following inclusion criteria: age ≥18 years of age with stably maintained HbA_1c_ levels of <0.5% fluctuation across 6 months of measurements and available laboratory data for HbA_1c_ and GA prior to a standardized liquid meal test [Ensure, Meiji Dairies Corporation, Tokyo, Japan; 500 kcal, 17.5 g fat (31.5%), 68.5 g carbohydrate (54.5%), and 17.5 g protein (14.0%)] after an overnight fast. Patients were excluded for the following reasons: any medical conditions that could alter HbA_1c_ or GA levels, including liver cirrhosis or kidney diseases (nephrotic syndrome or serum creatinine level ≥176.8 µM), pregnancy, steroid therapy, and hematologic disorders. The protocol of this study was approved by the Institutional Review Board at Severance Hospital (IRB No. 4-2009-0656, 4-2012-0398, 4-2013-0103, 4-2014-0507) and written informed consent for this study was not required by the Institutional Review Board because researchers only accessed the database for analysis purposes, and personal information was not used.

### Anthropometric and laboratory measurements

Using electronic medical records, demographic and clinical data were retrospectively collected for age, gender, smoking history, and duration of diabetes. The duration of diabetes was defined by the date when patients were diagnosed as having diabetes by blood tests or by patient recall from interviews. Body mass index (BMI) was calculated as weight in kilograms divided by the square of the height in meters. During standardized liquid meal tests, blood samples were collected at 0 and 90 min (basal and stimulated levels, respectively) for glucose, insulin, and C-peptide analyses. Serum C-peptide levels were determined by an immunoradiometric assay method (Beckman Coulter, Fullerton, CA, USA). Pancreatic β-cell function and insulin sensitivity were assessed using the following indices: homeostasis model assessment of pancreatic β-cell function (HOMA-β) = basal insulin (pM)×0.48/[basal glucose (mM)−3.5], HOMA-IR = [basal insulin (pM)×glucose (mM)/156.3] [12] and C-peptide increment (ΔC-peptide = C-peptide, stimulated−C-peptide, basal) [13], respectively. Serum concentrations of fasting glucose, total cholesterol, and albumin were measured by standard methods. Serum creatinine levels were determined with a Hitachi 7600-110 automated chemistry analyzer (Hitachi Co., Tokyo, Japan) with CREA (Roche Diagnostics, Indianapolis, IN, USA). Serum GA was analyzed by an enzymatic method using an albumin-specific proteinase, ketoamine oxidase, and an albumin assay reagent (LUCICA GA-L; Asahi Kasei Pharma Co., Tokyo, Japan), and a Hitachi 7699 P module autoanalyzer (Hitachi Instruments Service, Tokyo, Japan) [14]. GA values (%) were the calculation of the ratio of GA to total serum albumin. The coefficient of variation was 1.43%. HbA_1c_ was measured by high-performance liquid chromatography using Variant II Turbo (Bio-Rad Laboratories, Hercules, CA, USA). The reference ranges of HbA_1c_ and GA were between 4.0% and 6.0%, and between 11.0% and 16.0%, respectively.

### Statistical Analyses

All continuous variables were expressed as mean ± standard deviation (SD) or median (ranges) values, as appropriate. HOMA-β, HOMA-IR, and ΔC-peptide were log transformed for the analyses because the value distributions were skewed. Student's *t* test and Pearson's χ^2^ test were used to compare variables between two groups, as appropriate. The differences in GA and GA/HbA_1c_ ratios, between two groups, were evaluated using Student's *t* test with Bonferroni correction after stratification of HbA_1c_ levels, because GA/HbA_1c_ ratios are affected by HbA_1c_ levels [10]. One-way analysis of variance (ANOVA) was used to examine the differences of GA/HbA_1c_ ratios, GA, and ΔC-peptide levels according to the duration of diabetes or tertiles of ΔC-peptide. We analyzed the relationship between GA/HbA_1c_ ratios and ΔC-peptide, using Pearson's correlation coefficients with scatter plots. A spline curve was plotted for the relationship between GA and ΔC-peptide levels. A multivariable linear regression model was applied to assess various clinical and laboratory parameters associated with HbA_1c_ or GA. HOMA-IR was removed from the final linear model because it had multiple collinearity with basal insulin. Results were expressed as values of standardized β coefficient and *p*. To minimize the possible effect of age on GA, propensity-score matching was used to match ages among subjects. A two-sided *p* value <0.05 was considered significant. Statistical analyses were carried out with SPSS version 20.0 for Windows (IBM Corp., Armonk, NY, USA) and SAS version 9.2 (SAS Institute).

## Results

### Study population characteristics

New-T2D of 1059 subjects, defined as ≤1 year of diabetes duration, and 781 subjects with Old-T2D defined as >1 year of diabetes duration, were included in the present study. The patient characteristics of the cohort are shown in Table 1. Median durations of diabetes were 0.5 year and 4.4 years in New-T2D and Old-T2D, respectively. Individuals in the New-T2D group were younger and had lower levels of glucose at 90 min, while they had significantly higher values of stimulated C-peptide, ΔC-peptide, and total cholesterol. While HbA_1c_ levels were similar in the two groups (7.8±1.9 *vs.* 7.9±1.6, *p* = 0.159, respectively), both GA (19.6±7.8 *vs.* 20.9±7.4, *p*<0.001, respectively) and GA/HbA_1c_ ratios (2.47±0.50 *vs.* 2.61±0.53, *p*<0.001, respectively) were significantly increased in Old-T2D subjects, when compared to the New-T2D subjects.

**Table 1:** Baseline characteristics of the study population.

	New T2D	Old T2D	P
	diabetes duration ≤1 y (N = 1059)	diabetes duration >1 y (N = 781)	
Age (years)	56.8±12.3	60.7±11.3	<0.001
Sex (M/F, %Female)	625/434 (41)	435/346 (44)	0.154
Duration of diabetes (years)	0.5 (0–0.99)	4.4 (1.00–43.44)	<0.001
BMI (kg/m^2^)	25.3±3.6	25.1±3.7	0.486
Smoking (never/past/current)	645/224/190	502/139/140	0.184
**Glycemic profiles**			
Glucose, basal (mM)	7.6±2.7	7.5±2.5	0.768
Glucose, stimulated (mM)	12.2±4.9	12.8±4.4	0.005
HbA_1c_ (%)	7.8±1.9	7.9±1.6	0.159
HbA_1c_ (mM/M)	61.6±20.3	62.8±17.0	0.159
Glycated albumin (%)	19.6±7.8	20.9±7.4	<0.001
GA/HbA_1c_ ratio	2.47±0.50	2.61±0.53	<0.001
C-peptide, basal (nM)	0.82±0.45	.79±0.43	0.188
C-peptide, stimulated (nM)	2.10±1.03	1.90±0.94	<0.001
ΔC-peptide (nM)*	1.29±0.87	1.11±0.76	<0.001
Insulin, basal (pM)	82.8±91.1	83.5±97.2	0.903
Insulin, stimulated (pM)	400.1±355.2	348.6±280.6	0.003
HOMA-IR*	3.5±4.4	3.9±5.1	0.097
HOMA-β*	85.4±188.2	82.6±160.7	0.776
**Biochemistry profiles**			
Total cholesterol (mM)	4.7±1.2	4.3±1.0	<0.001
HDL cholesterol (mM)	1.2±0.3	1.2±0.3	0.660
LDL cholesterol (mM)	2.8±1.0	2.4±0.8	<0.001
Albumin (g/L)	45.0±3.7	45.1±3.4	0.763
Creatinine (µM)	81.8±20.5	86.0±23.1	<0.001

*log transformed.

Variables were described as mean ± SD or median (ranges).

BMI, body mass index; HOMA-IR, homeostasis model assessment of insulin resistance; HOMA-β, homeostasis model assessment of pancreatic β-cell function.

### Glycated albumin levels were elevated in subjects with long duration of diabetes

**Figure 1:**
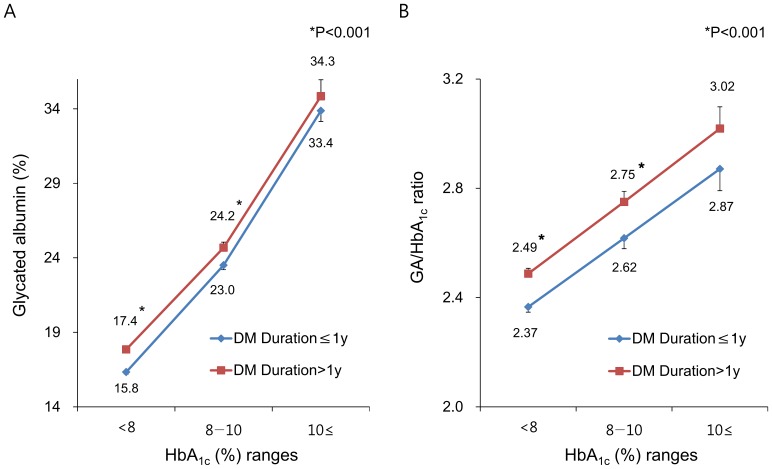
Differences of glycated albumin (GA) and GA/HbA_1c_ ratios according to the duration of diabetes by HbA_1c_ ranges. (**A**) glycated albumin; (**B**) GA/HbA_1c_ ratio. Data are shown as mean with SD (bars). *P<0.001 compared to those from subjects with New-T2D.

Based on recent studies, the levels of GA and GA/HbA_1c_ ratios were higher in subjects with poorly controlled diabetes than in subjects with well-controlled diabetes, whereas HbA_1c_ levels were not different [10]. Likewise, patients with longer diabetes duration tend to have higher HbA_1c_ levels [15]. Therefore, GA and GA/HbA_1c_ ratios in this study were compared, according to HbA_1c_ strata (Fig. 1). In the ranges of HbA_1c_ levels <8%, and 8 to 10%, both GA and GA/HbA_1c_ ratios in Old-T2D were significantly higher than in New-T2D. Average GA values in Old-T2D subjects were 0.9 to 1.6% higher than in New-T2D subjects.

**Figure 2:**
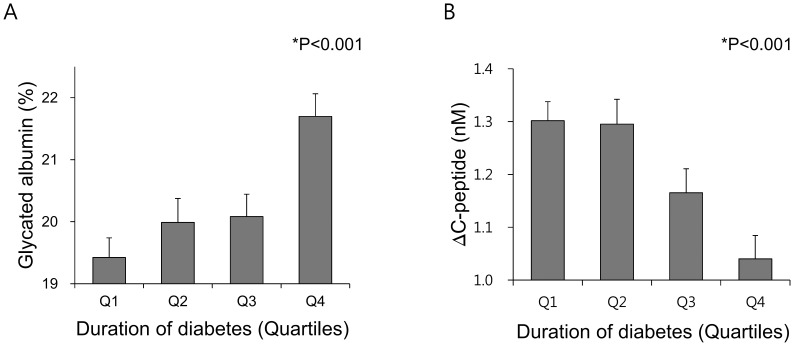
Association between duration of diabetes and glycated albumin or C-peptide increment (ΔC-peptide). (**A**) Difference of glycated albumin levelsaccording to the duration of diabetes; (**B**) Difference of ΔC-peptide levels according to the duration of diabetes. Data are shown as mean with SD (bars).

To assess the association between GA-related indices and diabetes duration, the duration of diabetes was categorized into quartiles. Consistent with the results shown in Fig. 1, GA (Fig. 2A) and GA/HbA_1c_ ratios (Fig. S1) were significantly and sequentially increased across the quartiles of diabetes duration. However, as diabetes duration increased, the level of ΔC-peptide, an insulin secretory index of β-cells, was statistically decreased (Fig. 2B), indicating the gradual deterioration of reserve β-cell function over time.

### Glycated albumin was negatively correlated with insulin secretory indices

**Figure 3:**
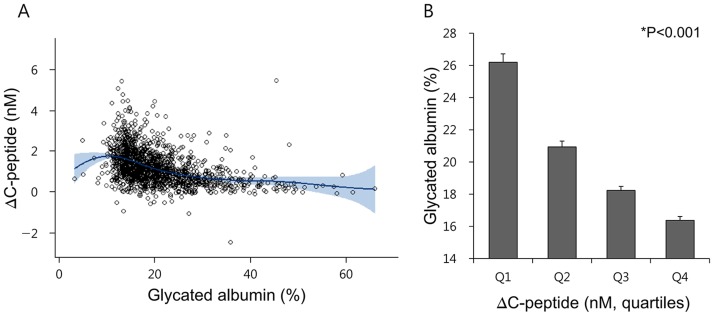
Association between glycated albumin and C-peptide increment (ΔC-peptide). (**A**) A plot of spline curve for association between glycated albumin and ΔC-peptide levels. Data plotted by a spline curve (dark blue) and 95% confidence interval (light blue); (**B**) Difference of glycated albumin levels according to the ΔC-peptide levels.

Considering the detrimental effects of diabetes duration on GA/HbA_1c_ ratio and ΔC-peptide levels, correlation analysis was performed to assess the link between GA-related indices and parameters of β-cell functions (Table 2 and Fig. 3). Simple and partial correlation analyses both showed that GA levels were negatively related to insulin secretory indices such as HOMA-β, Δinsulin, and basal C-peptide, whereas basal insulin levels had no significant correlation with GA (Table 2 and Fig. S2). Duration of diabetes was also positively related to GA levels (*r* = 0.12, *p*<0.001), regardless of age. Strong inverse correlation was found between ΔC-peptide levels and GA (*r* = −0.41, *p*<0.001 in Table 2, or Fig. 3A) or GA/HbA_1c_ ratios (*r* = −0.35, *p*<0.001, Fig. S3), regardless of age and gender. Values of both GA (Fig. 3B) and GA/HbA_1c_ ratios (Fig. S3) were gradually decreased as quartiles of ΔC-peptide levels increased.

**Table 2:** Correlation analyses to determine the association between glycated albumin and other variables including insulin secretory indices and the duration of diabetes.

	Glycated albumin			
	Simple correlation		Partial correlation*	
	r	P	r	P
Age (years)	−0.010	0.680	–	–
BMI (kg/m^2^)	−0.185	<0.001	−0.200	<0.001
Glucose, basal (mM)	0.614	<0.001	0.670	<0.001
Glucose, stimulated (mM)	0.676	<0.001	0.704	<0.001
Total cholesterol (mM)	0.033	0.181	0.005	0.878
HOMA-IR	0.186	<0.001	0.201	<0.001
HOMA-β	−0.072	0.006	−0.120	<0.001
Insulin, basal (pM)	0.026	0.314	0.029	0.355
ΔInsulin (pM)	−0.295	<0.001	−0.318	<0.001
C-peptide, basal (nM)	−0.148	<0.001	−0.128	<0.001
ΔC-peptide (nM)	−0.412	<0.001	−0.442	<0.001
Albumin (g/L)	−0.091	<0.001	−0.108	<0.001
Creatinine (µM)	0.026	0.306	−0.005	0.882
Duration of diabetes (years)	0.123	<0.001	0.120	<0.001

Partial correlation* was conducted with adjustment for age and sex.

### The association between glycated albumin and C-peptide increment (ΔC-peptide) accounted for increased levels of glycated albumin in subjects with longer duration of diabetes

Multiple linear regression models were applied to determine the clinical and laboratory parameters associated with HbA_1c_ or GA (Table 3). In model 1, which included clinically and statistically important variables except ΔC-peptide levels, duration of diabetes had a positive correlation with both GA (standardized [STD] β = 0.05, *p* = 0.02) and GA/HbA_1c_ ratio (STD β = 0.05, *p* = 0.03, Table S1), but not with HbA_1c_ levels (STD β = 0.04, *p* = 0.10). Contrary to glucose profiles, both age and serum albumin levels were negatively correlated with HbA_1c_ levels, while BMI, total cholesterol, and albumin levels were negatively correlated with GA (model 1). After additional adjustment for ΔC-peptide levels in the existing model (model 2), the significant correlation of duration of diabetes with GA (STD β = 0.02, *p* = 0.37) or with GA/HbA_1c_ ratio (STD β = 0.02, *p* = 0.43, Table S1) disappeared. However, ΔC-peptide level was strongly associated with both GA and GA/HbA_1c_ ratio (STD β = −0.22 and −0.21, respectively; both *p*<0.001), which led to increments of R^2^ values (from 0.36 in Model 1 to 0.40 in Model 2). These findings remained consistent in further analysis with age-matched study population (Table S2).

**Table 3:** Multiple linear regression analyses to determine the variables associated with HbA1c or glycated albumin.

	HbA_1c_				Glycated albumin			
	Model 1		Model 2		Model 1		Model 2	
	STD β	P	STD β	P	STD β	P	STD β	P
Age (years)	−0.087	<0.001	−0.044	0.075	−0.010	0.654	0.046	0.040
Sex (F = 0, M = 1)	−0.030	0.329	−0.010	0.751	−0.022	0.428	0.006	0.825
BMI (kg/m^2^)	0.005	0.828	0.024	0.317	−0.113	<0.001	−0.073	0.001
Smoking (never = 0, ever = 1)	0.013	0.633	0.012	0.661	−0.010	0.679	−0.013	0.579
Glucose, basal (mM)	0.300	<0.001	0.236	<0.001	0.351	<0.001	0.305	<0.001
Glucose, stimulated (mM)	0.428	<0.001	0.414	<0.001	0.419	<0.001	0.392	<0.001
Total cholesterol (mM)	−0.001	0.978	0.012	0.599	−0.050	0.018	−0.038	0.069
Insulin, basal (pM)	0.049	0.035	0.028	0.240	0.070	0.001	0.038	0.079
C-peptide, basal (nM)	−0.051	0.042	0.009	0.735	−0.130	<0.001	−0.075	0.002
Albumin (g/L)	−0.113	<0.001	−0.091	<0.001	−0.038	0.064	−0.006	0.760
Creatinine (µM)	−0.030	0.250	−0.056	0.042	0.049	0.046	0.013	0.605
**Duration of diabetes (years)**	**0.037**	**0.095**	**0.022**	**0.330**	**0.047**	**0.023**	**0.018**	**0.372**
**ΔC-peptide (nM)** *	**–**	**–**	**−0.196**	**<0.001**	**–**	**–**	**−0.223**	**<0.001**

*log transformed.

STD β, standardized β coefficient; BMI, body mass index.

## Discussion

**Figure 4:**
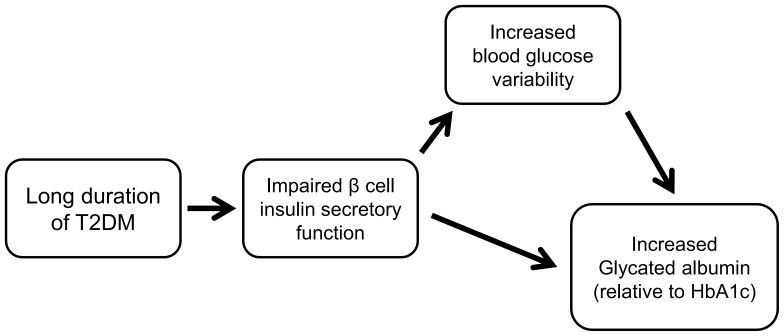
A putative diagram of the relationship among glycated albumin, β-cell function and duration of diabetes.

The present study observed that GA levels or GA/HbA_1c_ ratios were elevated in Old-T2D subjects than in New-T2D subjects, regardless of their glycemic control status (HbA_1c_ levels). With respect to insulin-related parameters, GA was negatively correlated with dynamic insulin secretory indices, but not with basal insulin levels. In addition, GA, but not HbA_1c_ was significantly associated with duration of diabetes. This association did not remain after additional adjustment with ΔC-peptide in the regression model, indicating that insulin secretory function played a major role in this close relationship between GA and duration of diabetes. Although this is a cross-sectional study which cannot draw any causal relationships, we may suggest that GA values are indirectly associated with the long duration of T2D, because β-cell function gradually decreases as duration of diabetes increases (Fig. 4). Impaired insulin secretion from β-cells can increase blood glucose excursions, which is more sensitively reflected by GA compared to HbA_1c_.

HbA_1c_ levels depend on glucose transport from plasma into erythrocytes and on intracellular glucose and protein metabolism, which indirectly reflect glycemic status. However, GA is not affected by extracellular–intracellular glucose dynamics [16], but directly produced by the glycation process in the plasma. In addition, GA level is not changed by serum concentration of albumin, because it is calculated by the glycated proportion of total serum albumin [17]. However, several factors can influence GA values. It is well known that obesity (BMI) is inversely associated with GA [18], which is consistent with our regression analyses. This result may be due to increased albumin catabolism induced by chronic micro-inflammatory conditions in obese subjects [19]. Likewise, GA levels are more inaccurate compared to HbA_1c_ values in certain diseases affecting albumin metabolism [17]. During hyper-metabolic states of albumin, such as nephrotic syndrome, hyperthyroidism, and glucocorticoid treatment, GA increases in relation to blood glucose, while it decreases in subjects with diminished albumin catabolism, including liver cirrhosis and hypothyroidism [17]. To minimize the effects of erythrocytes or albumin turnover on HbA_1c_ or GA values, we excluded subjects who had liver cirrhosis or CKD, pregnancy, steroid therapy, and hematologic disorders such as anemia.

In addition to the above listed pathologic conditions, the present study showed that GA or GA/HbA_1c_ ratios were significantly increased in subjects with longer duration of T2D. Duration of diabetes was indirectly associated with GA and decreased β-cell function might be the linking factor between duration of diabetes and GA values. This was supported by our results from linear regression analyses showing that diabetic duration was associated with GA, of which association disappeared after adjustment with β-cell function index (ΔC-peptide). Among various insulin secretory indices, static parameters such as basal insulin or HOMA-β showed no or minimal relationships with GA, whereas dynamic parameters such as ΔC-peptide or Δinsulin had strong correlation with GA. To date, it is still not known why GA is closely correlated with endogenous insulin secretory function. GA is known to function as a pathogenic protein as well as an index for glycemic status, because it is an early precursor of advanced glycation end-products (AGE), which cause alterations in various cellular proteins and organelles, leading to apoptosis [5]. A recent report suggested that GA induced pancreatic β-cell dysfunction and death by disrupting cellular defense homeostasis [20]. Similarly, GA directly suppressed glucose-stimulated insulin secretion from rat pancreatic β-cells through impairment of intracellular glucose metabolism [21]. These may support the inverse relationship found between GA and β-cell function, because increased circulating levels of GA can lead to secretory dysfunction in β-cells, a pathophysiology known as glucolipotoxicity. In addition, considering the role of GA as a short-term (3-week) glycemic index, GA may reflect glucose fluctuation and postprandial glucose more sensitively than HbA_1c_ [10], [22]–[24].

Our study has some distinct strengths. This was a rather large population-based study consisting of both newly diagnosed and long duration of T2D subjects, which could detect mild association of diabetes duration with GA and enhance the statistical reliability of the results. Our findings that GA is significantly elevated relative to HbA_1c_ in certain patients with T2D, may raise serious issues regarding GA as an adequate index for monitoring glycemic control, especially in subjects with longer duration of diabetes, or severely impaired endogenous insulin secretion, such as during type 1 diabetes. Consistent with our results, Koga et al. reported that GA/HbA_1c_ ratios were elevated in T2D patients treated with insulin, as well as in autoimmune acute-onset type 1 diabetic patients [8], [25]. This was attributed to strong links between GA and β-cell function. Because GA is less consistent in predicting blood glucose levels among subjects who have newly-diagnosed or long-standing T2D compared to HbA_1c_, clinicians should be cautious when using GA as a substitute index for monitoring glycemic status, especially in terms of long-term follow-up. Although GA is a superior index to HbA_1c_ for subjects with anemia or CKD, duration of diabetes as well as insulin secretory function should be taken into consideration when interpreting GA values.

The current study has several potential limitations, which should be addressed by future research. First, the cross-sectional approach did not allow us to draw complete conclusions regarding time-dependent changes of GA and GA/HbA_1c_ ratios in subjects with T2D or with transition in β-cell function. Second, assessment of endogenous insulin secretory function was confined to C-peptide-related indices. Due to lack of data from oral glucose tolerance tests, various insulin secretory parameters were not analyzed in this study. Third, other factors such as anti-diabetic medication, which may affect the variation in GA levels, were not evaluated. Lastly, duration of diabetes was unable to be assessed precisely in each individual due to slow-progressive characteristics of T2D, but was clinically defined, similar to previous studies [26]. Therefore, its pathognomic meaning is feeble.

In conclusion, the present study clearly showed that GA/HbA_1c_ ratios were significantly elevated in subjects with longer duration of T2D, largely due to inverse relationships between GA and β-cell secretory indices. Clinicians should be careful in interpreting GA values as assessment indices of glycemic control in these individuals. Future investigation is warranted to elucidate the mechanism of the negative association between GA and β-cell function, and to validate the role of GA as a reliable index for long-term glycemic monitoring in various clinical conditions.

## Supporting Information

Figure S1 Difference of GA/HbA_1c_ ratios according to the duration of diabetes.
(DOCX)

Figure S2 **Association between glycated albumin and insulin or C-peptide-related parameters.** Correlation analysis of glycated albumin with basal insulin levels (A); Δinsulin levels (stimulated insulin – basal insulin) (B); HOMA-β (C).
(DOCX)

Figure S3 **Correlation analysis of GA/HbA_1c_ ratios with ΔC-peptide levels (A); Difference of GA/HbA_1c_ ratios according to the ΔC-peptide levels (B).**
(DOCX)

Table S1 **Multiple linear regression analyses to determine the variables associated with GA/HbA_1c_ ratio.**
(DOCX)

Table S2 **Multiple linear regression analyses to determine the variables associated with HbA_1c_ or glycated albumin in age-matched study cohort (N = 1562).**
(DOCX)
