# Supplementary figures and images for: Inverse Association between Glycated Albumin and Insulin Secretory Function May Explain Higher Levels of Glycated Albumin in Subjects with Longer Duration of Diabetes
Source: PLoS One. 2014 Sep 29;9(9):e108772. doi: 10.1371/journal.pone.0108772 (PMC4181354; doi:10.1371/journal.pone.0108772)

**Figure S1.** Difference of GA/HbA1c ratios according to the duration of diabetes.


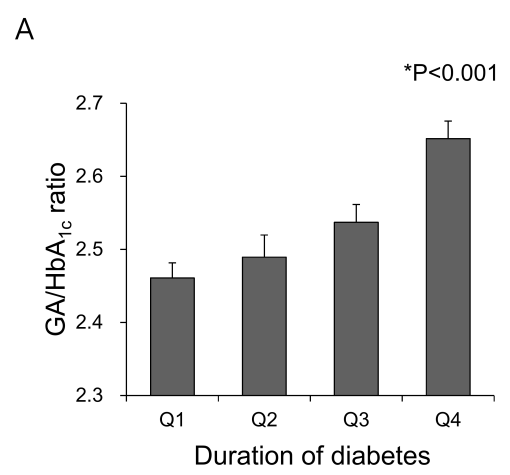

Supplement: Figure S1 — Difference of GA/HbA1c ratios according to the duration of diabetes. (DOCX) [file pone.0108772.s001.docx]
