# Supplementary material for: Inverse Association between Glycated Albumin and Insulin Secretory Function May Explain Higher Levels of Glycated Albumin in Subjects with Longer Duration of Diabetes
Source: PLoS One. 2014 Sep 29;9(9):e108772. doi: 10.1371/journal.pone.0108772 (PMC4181354; doi:10.1371/journal.pone.0108772)

**Figure S2.** Association between glycated albumin and insulin or C-peptide-related parameters. Correlation analysis of glycated albumin with basal insulin levels (A); ∆insulin levels (stimulated insulin – basal insulin) (B); HOMA-β (C).


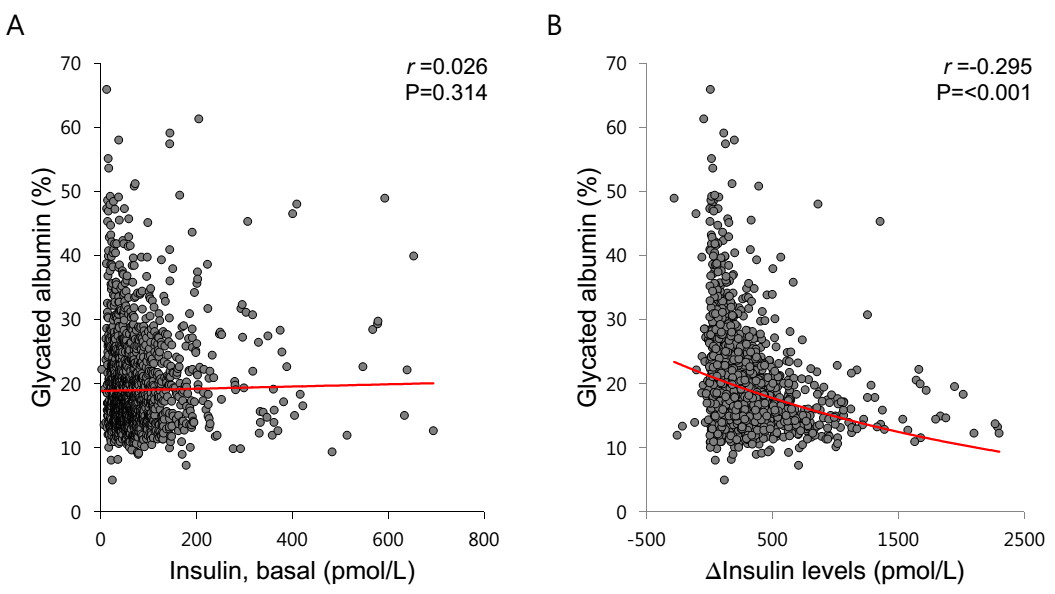


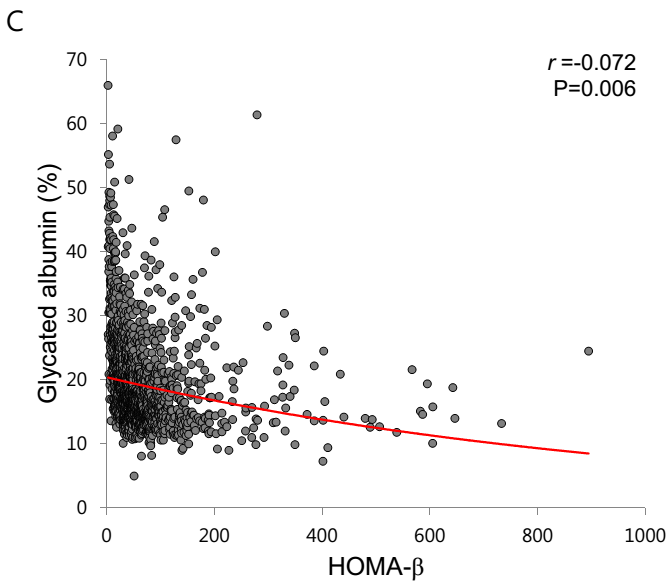

Supplement: Figure S2 — Association between glycated albumin and insulin or C-peptide-related parameters. Correlation analysis of glycated albumin with basal insulin levels (A); Δinsulin levels (stimulated insulin – basal insulin) (B); HOMA-β (C). (DOCX) [file pone.0108772.s002.docx]
