# Supplementary material for: Inverse Association between Glycated Albumin and Insulin Secretory Function May Explain Higher Levels of Glycated Albumin in Subjects with Longer Duration of Diabetes
Source: PLoS One. 2014 Sep 29;9(9):e108772. doi: 10.1371/journal.pone.0108772 (PMC4181354; doi:10.1371/journal.pone.0108772)

**Figure S3.** Correlation analysis of GA/HbA_1c_ ratios with ∆C-peptide levels (A); Difference of GA/HbA_1c_ ratios according to the ∆C-peptide levels (B).


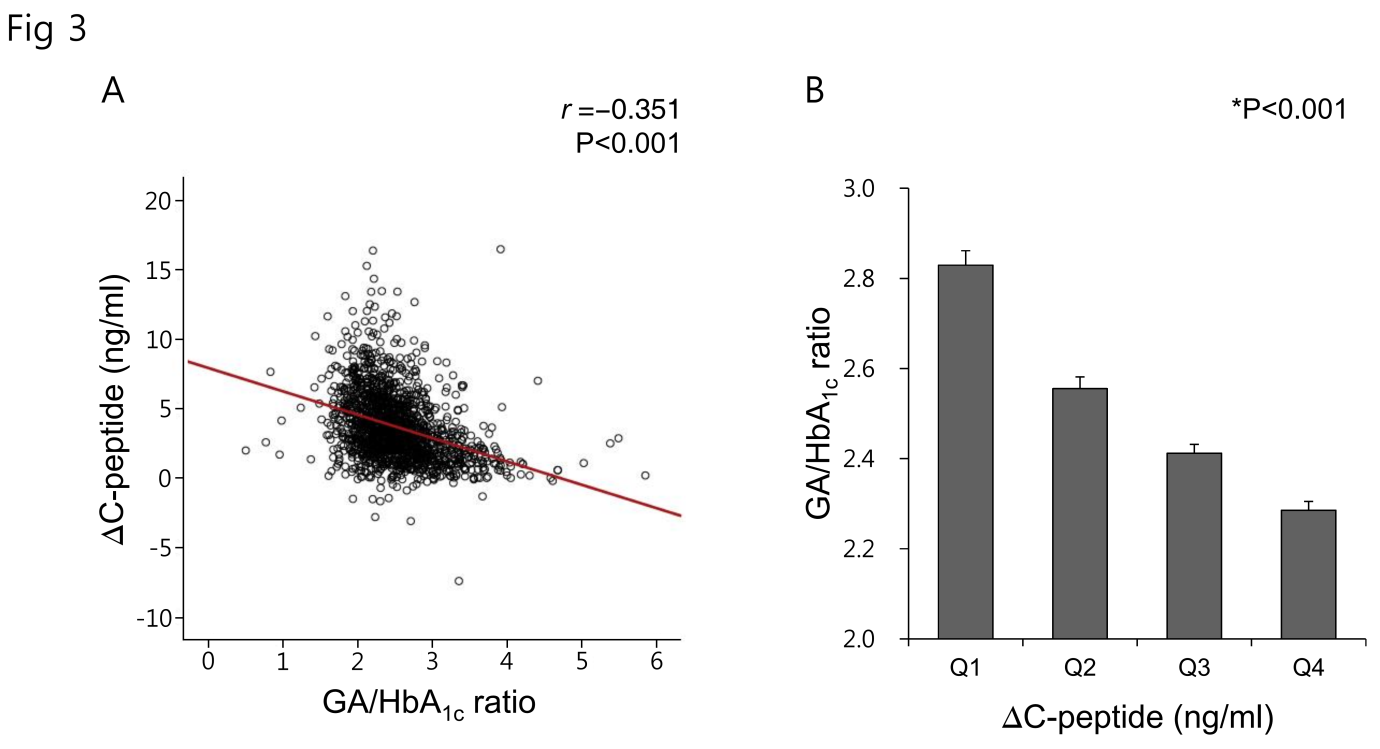

Supplement: Figure S3 — Correlation analysis of GA/HbA1c ratios with ΔC-peptide levels (A); Difference of GA/HbA1c ratios according to the ΔC-peptide levels (B). (DOCX) [file pone.0108772.s003.docx]
