# Supplementary material for: Inverse Association between Glycated Albumin and Insulin Secretory Function May Explain Higher Levels of Glycated Albumin in Subjects with Longer Duration of Diabetes
Source: PLoS One. 2014 Sep 29;9(9):e108772. doi: 10.1371/journal.pone.0108772 (PMC4181354; doi:10.1371/journal.pone.0108772)
Supplement: Table S1 — Multiple linear regression analyses to determine the variables associated with GA/HbA1c ratio. (DOCX) [file pone.0108772.s004.docx]

**Table S1.** Multiple linear regression analyses to determine the variables associated with GA/ HbA_1c_ ratio.

|  | **GA/HbA_1c_ ratio** | | | | |  |
| --- | --- | --- | --- | --- | --- | --- |
|  | **Model 1** | |  | **Model 2** | |  |
|  | **STD β** | **P** |  | **STD β** | **P** |  |
| Age (years) | 0.099 | <0.001 |  | 0.151 | <0.001 |  |
| Sex (F=0, M=1) | 0.015 | 0.656 |  | 0.037 | 0.283 |  |
| BMI (kg/m^2^) | -0.192 | <0.001 |  | -0.144 | <0.001 |  |
| Smoking (never=0, ever=1) | -0.043 | 0.148 |  | -0.047 | 0.108 |  |
| Glucose, basal (mM) | 0.270 | <0.001 |  | 0.224 | <0.001 |  |
| Glucose, stimulated (mM) | 0.284 | <0.001 |  | 0.262 | <0.001 |  |
| Total cholesterol (mM) | -0.080 | 0.002 |  | -0.070 | 0.006 |  |
| Insulin, basal (pM) | 0.061 | 0.021 |  | 0.025 | 0.336 |  |
| C-peptide, basal (nM) | -0.171 | <0.001 |  | -0.123 | <0.001 |  |
| Albumin (g/L) | 0.027 | 0.282 |  | 0.065 | 0.010 |  |
| Creatinine (μM) | 0.102 | 0.001 |  | 0.068 | 0.026 |  |
| **Duration of diabetes (years)** | **0.053** | **0.030** |  | **0.020** | **0.427** |  |
| **ΔC-peptide (nM)*** | **−** | **−** |  | **-0.210** | **<0.001** |  |

*log transformed.

STD β, standardized β coefficient; BMI, body mass index.
