# Supplementary material for: Inverse Association between Glycated Albumin and Insulin Secretory Function May Explain Higher Levels of Glycated Albumin in Subjects with Longer Duration of Diabetes
Source: PLoS One. 2014 Sep 29;9(9):e108772. doi: 10.1371/journal.pone.0108772 (PMC4181354; doi:10.1371/journal.pone.0108772)
Supplement: Table S2 — Multiple linear regression analyses to determine the variables associated with HbA1c or glycated albumin in age-matched study cohort (N = 1562). (DOCX) [file pone.0108772.s005.docx]

**Table S2.** Multiple linear regression analyses to determine the variables associated with HbA_1c_ or glycated albumin in age-matched study cohort (N=1562)

|  | **HbA_1c_** | | | | |  | **Glycated albumin** | | | | |
| --- | --- | --- | --- | --- | --- | --- | --- | --- | --- | --- | --- |
|  | **Model 1** | |  | **Model 2** | |  | **Model 1** | |  | **Model 2** | |
|  | **STD β** | **P** |  | **STD β** | **P** |  | **STD β** | **P** |  | **STD β** | **P** |
| Age (years) | -0.085 | 0.001 |  | -0.056 | 0.041 |  | 0.013 | 0.599 |  | 0.052 | 0.035 |
| Sex (F=0, M=1) | -0.023 | 0.497 |  | -0.020 | 0.567 |  | -0.010 | 0.750 |  | -0.009 | 0.786 |
| BMI (kg/m^2^) | 0.007 | 0.783 |  | 0.017 | 0.533 |  | -0.104 | <0.001 |  | -0.073 | 0.003 |
| Smoking (never=0, ever=1) | 0.023 | 0.448 |  | 0.026 | 0.381 |  | -0.005 | 0.859 |  | -0.002 | 0.952 |
| Glucose, basal (mM) | 0.309 | <0.001 |  | 0.240 | <0.001 |  | 0.378 | <0.001 |  | 0.320 | <0.001 |
| Glucose, stimulated (mM) | 0.394 | <0.001 |  | 0.389 | <0.001 |  | 0.392 | <0.001 |  | 0.376 | <0.001 |
| Total cholesterol (mM) | <0.001 | 0.992 |  | 0.007 | 0.769 |  | -0.045 | 0.053 |  | 0-.040 | 0.081 |
| Insulin, basal (pM) | 0.052 | 0.048 |  | 0.033 | 0.219 |  | 0.075 | 0.002 |  | 0.046 | 0.054 |
| C-peptide, basal (nM) | -0.040 | 0.158 |  | 0.012 | 0.674 |  | -0.126 | <0.001 |  | -0.088 | 0.001 |
| Albumin (g/L) | -0.125 | <0.001 |  | -0.113 | <0.001 |  | -0.057 | 0.013 |  | -0.033 | 0.155 |
| Creatinine (μM) | -0.042 | 0.161 |  | -0.049 | 0.108 |  | 0.042 | 0.121 |  | 0.034 | 0.215 |
| **Duration of diabetes (years)** | **0.041** | **0.095** |  | **0.020** | **0.412** |  | **0.053** | **0.019** |  | **0.016** | **0.470** |
| **ΔC-peptide (nM)*** | **−** | **−** |  | **-0.196** | **<0.001** |  | **−** | **−** |  | **-0.230** | **<0.001** |

*log transformed.

STD β, standardized β coefficient; BMI, body mass index.
